# SARS Exposure and Emergency Department Workers

**DOI:** 10.3201/eid1006.030972

**Published:** 2004-06

**Authors:** Wei-Tien Chang, Chuan-Liang Kao, Ming-Yi Chung, Shyr-Chyr Chen, Shou-Ju Lin, Wen-Chu Chiang, Shey-Ying Chen, Chan-Ping Su, Po-Ren Hsueh, Wen-Jone Chen, Pei-Jer Chen, Pan-Chyr Yang

**Affiliations:** * National Taiwan University Hospital and College of Medicine, Taipei, Taiwan

**Keywords:** SARS, serologic responses, emergency department workers

## Abstract

Of 193 emergency department workers exposed to severe acute respiratory syndrome (SARS), 9 (4.7%) were infected. Pneumonia developed in six workers, and assays showed anti-SARS immunoglobulin (Ig) M and IgG. The other three workers were IgM-positive and had lower IgG titers; in two, mild illness developed, and one remained asymptomatic.

The first case of severe acute respiratory syndrome (SARS) in Taiwan was reported from the National Taiwan University Hospital (NTUH) in mid-March 2003 (*1*). An infected businessman returning from mainland China was the source of a cluster of infections involving his family and a physician. Thereafter, a number of sporadic cases or small outbreaks emerged in the following month, mostly imported from abroad.

A tertiary university medical center in metropolitan Taipei, NTUH was responsible for most SARS screening during this time. Many patients with symptoms or signs of SARS were transferred to the emergency department of NTUH for evaluation and management. After April 20, 2003, the number of SARS patients increased markedly because of outbreaks in two hospitals in Taipei. During the epidemic in Taiwan, >2,000 febrile patients visited the emergency department of NTUH, and laboratory-confirmed SARS was diagnosed in 79 of them. All 79 patients tested positive for anti-SARS immunoglobulin (Ig) G by using a commercial immunofluorescent assay (IFA) (EUROIMMUN Anti-SARS-CoV-IIFT, Lübeck, Germany), and 25 of them also had positive results of reverse transcriptase–polymerase chain reaction on two separate respiratory samples (sputum or throat swabs) or one respiratory sample and one nonrespiratory (urine or stool) sample. After exposure to SARS, fever or diarrhea occurred in many emergency department workers, and 13 of them were admitted to the hospital. On May 12, the emergency department of NTUH was closed; it was reopened on May 26, when all personnel had no indications of disease after >10 days of isolation.

## The Study

Clinical symptoms and signs (fever, cough, headache, sore throat, rhinorrhea, and diarrhea), which developed in the 193 healthcare workers working at the emergency department of NTUH from March 30 to June 30, were retrospectively evaluated through a formal questionnaire. Two IFA methods (in-house and EUROIMMUN) for detecting IgG, IgM, and IgA, and a direct enzyme-linked immunosorbent assay (ELISA) (SARS-96[TNB], General Biologicals Corp, Hsin-Chu, Taiwan) for detecting IgG against the SARS–associated coronavirus (SARS-CoV) were performed on serum specimens from all of these workers. For in-house IFA, whole-cell lysate of infected Vero E6 cells was used as an antigen. Spot slides were prepared by applying the suspension mixed with Vero E6 cells infected with SARS-CoV (TW1 strain, GenBank accession no. AY291451) and uninfected cells. Slides were dried and fixed in acetone. The conjugates used were goat antihuman IgG, IgM, and IgA conjugated to fluorescein isothiocyanate (Organon Teknika-Cappel, Turnhout, Belgium). The starting dilution of serum specimens was 1:25 for the in-house IFA and 1:10 for the EUROIMMUN kit. Before IgM and IgA were determined by IFA, antibodies of class IgG were removed from the patient's serum by antihuman IgG by using two immunoabsorption kits: EUROSORB (EUROIMMUN) for commercial IFA and GULLSORB (Meridian Bioscience Inc., Cincinnati, OH) for the in-house assay. The cutoff values for a positive result for IgG, IgM, and IgA were all 1:25 by in-house IFA and 1:10 by the commercial IFA kit. IgG against SARS-CoV by an indirect ELISA was also performed by using recombinant nucleocapsid as the coated antigen. The cutoff value of IgG by ELISA was 0.26.

As control sera, we used 200 paired samples from patients with community-acquired pneumonia seen at NTUH from October 2001 to December 2002, 70 serum samples from hospitalized patients with acute respiratory distress syndrome treated in 2002 at the hospital, and 10 serum samples obtained from 10 pregnant women during routine pre-labor check-ups in 2002. All control serum specimens were negative for IgG by ELISA and IgG, IgM, or IgA by two IFA methods.

Reverse transcriptase–polymerase chain reaction (RT-PCR) assays (nested and real-time) for respiratory specimens (throat swabs and sputum) and serum were performed for the six workers in whom pneumonia developed. Cases of laboratory-confirmed SARS in workers were further classified as asymptomatic, mild, or severe, according to the criteria provided by the Centers for Disease Control and Prevention (*2*).

The 193 workers included 54 men and 139 women, with a mean age of 32.7 ± 8.2 years. They included 33 physicians, 95 nurses, 17 radiology technicians, 16 clerks, 13 sanitation workers, 13 administrative personnel, and 6 ambulance drivers. From March 30 to June 30, 2003, 45 (25.4%) of these workers reported feeling feverish, 51 (26.4%) had cough, 47 (24.4%) had myalgia, 60 (31.6%) had headache, 54 (28.0%) had sore throat, 41 (21.2%) had rhinorrhea, and 52 (26.9%) had diarrhea. From May 8 to May 20, 13 of these workers were consecutively admitted and isolated; these included 3 physicians, 7 nurses, 2 sanitation workers, and 1 clerk. Six (3.1%, 6/193) of these 13 patients, including 3 nurses, 2 sanitation workers, and 1 clerk, met the criteria for severe SARS caused by pneumonia, positive RT-PCR results for respiratory specimens and serum samples (5 patients), and positive antibody responses. These six patients had a date of disease onset between May 10 and May 17. They were also positive for IgM and IgA and had high titers of specific IgG (1:800 to 1:3,200) against SARS-CoV (Figure, part A). The IgG antibody titers remained high for >150 days after illness onset. Two of the three physicians (patients A and B) had positive results for both IgM and IgG, although they both had only transient fever (38°C for <1 day) and chills without any respiratory illness (rhinorrhea, sore throat, cough, or dyspnea) or gastrointestinal symptoms (nausea, vomiting, or diarrhea); transient fever developed on May 11 in patient A and on May 12 in patient B. Their leukocyte and lymphocyte counts and liver function were normal. The IgG antibody titers in these two patients were low (1:25 to 1:100) and disappeared rapidly (52 days and 148 days, respectively, after onset of illness; Figure, part B) compared with those of patients with severe SARS, in whom IgG is still present as of this writing. RT-PCR studies (Artus, Roche Diagnostics, Hamburg, Germany) of these two patients' serum samples were negative for SARS-CoV. The other five workers who were admitted had negative antibody results; however, one of them (a nurse) was positive (1:640) for antibodies against *Mycoplasma pneumoniae* when the particle agglutination method was used.

**Figure:**
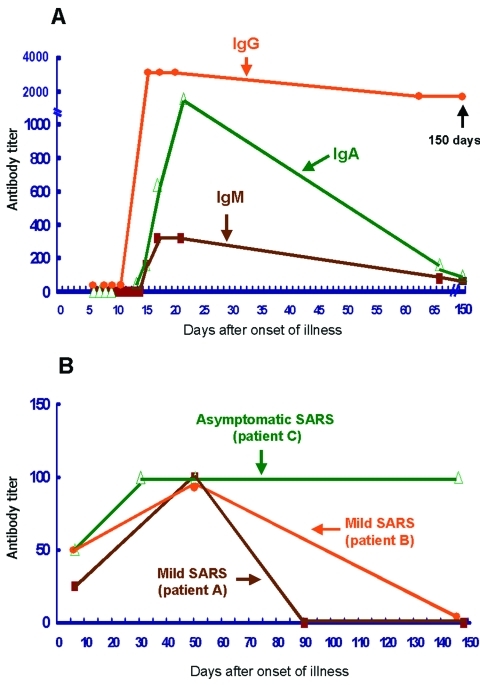
Changes over time in levels of antibodies against severe acute respiratory syndrome–associated coronavirus (SARS-CoV) in patients with laboratory-confirmed SARS. A denotes the changes in immunoglobulin (Ig) G, IgM, and IgA titers for a representative patient with severe SARS. B denotes the changes in IgG for two patients with mild SARS and one asymptomatic worker with SARS-CoV infection. The date of illness onset for patient C was assumed to be May 12, 2003 (the mean date of eight other SARS patients during the outbreak).

All but one (an ambulance driver, patient C) of the 180 workers who were not admitted had negative ELISA and IFA test results on serum samples collected in late June 2003. This worker had normal leukocyte and lymphocyte counts and did not show any signs or symptoms of respiratory illness. However, he had elevated IgM and IgG titers by IFA, which were both at detectable levels (1:10 and 1:50, respectively) on May 17. The IgM titer remained positive in serum samples collected on June 9 and then became negative on June 30 and was still negative on October 6. Results of RT-PCR studies (Artus, Roche Diagnostics) of the serum, throat swab, and stool specimens from this patient were negative.

Overall, the incidence of SARS-CoV infection among the emergency department workers in this hospital was 4.7% (9/193), including 6 (3.1%) with severe SARS, 2 (1.0%) with mild SARS, and 1 (0.6%) who was asymptomatic. The incidence of SARS-CoV infection was highest in ambulance drivers (16.7%), followed by sanitation workers (15.4%), clerks (6.3%), physicians (6.1%), and nurses (3.2%).

## Conclusions

This study illustrates three key aspects of the spread of SARS in an emergency department setting. First, not only the medical personnel but also the paramedical workers were at risk for SARS-CoV infection. Although universal precautions should be strictly followed when staff encounter patients with a variety of symptoms and signs, implementing infection-control measures is more difficult in the emergency department than in the wards or intensive care units, after patients' conditions have been identified. In fact, emergency department medical staff members have been reported to be at a higher risk for infection than staff members in other hospital departments (*3*). Second, persons infected with SARS-CoV might manifest only transient febrile illness and minimal respiratory illness or be completely free of any clinical symptoms or signs suggestive of SARS. These findings highlight the possibility that SARS-CoV might produce only mild or asymptomatic infection, although few previous reports have described this form of infection with SARS-CoV (*4**,**5*).

Finally, patients with mild or asymptomatic SARS-CoV infection in this study had lower levels (<1:100) of IgG antibody and earlier seroconversion than those of patients with severe SARS. This finding partly supports the hypothesis that an upsurge of antibody response is associated with increased severity of pulmonary condition (*1*). However, Lee et al. reported that a nurse with asymptomatic SARS-CoV infection had an IgG antibody titer as high as 1:400; IgG titers on the follow-up serum samples were not reported (*4*). Li et al. reported two cases of mild SARS, but antibody titers of these two patients were not reported (*5*). Serologic study of serial serum samples from more persons with mild illness or no symptoms is needed to confirm our findings of lower levels of IgG and earlier seroconversion.

Approximately 30% of emergency department workers without SARS-CoV infection in this study had clinical symptoms and signs similar to those of SARS during this epidemic. These illnesses might have been due to influenza or other upper airway infections; however, differentiating between SARS and other respiratory tract infections in these patients was difficult.

This study not only highlights the presence of mild and asymptomatic infection in healthcare workers during a SARS epidemic but also indicates lower antibody response and earlier seroconversion. Controlling this highly infective emerging disease requires meticulous preparation and vigilance by every worker in the emergency department.
